# Multi-Reference Evaluation of Deep Learning Tumour Segmentation in Multi-Sequence Nasopharyngeal Carcinoma MRI

**DOI:** 10.3390/diagnostics16213632

**Published:** 2026-11-03

**Authors:** Ema Pandilova, Marko Petrov, Ivan Chorbev, Ivan Kitanovski, Aleksandar Stojmenski, Dimitar Trajanov

**Affiliations:** Faculty of Computer Science and Engineering, Ss. Cyril and Methodius University in Skopje, 1000 Skopje, North Macedonia; marko.petrov@finki.ukim.mk (M.P.); ivan.chorbev@finki.ukim.mk (I.C.); ivan.kitanovski@finki.ukim.mk (I.K.); aleksandar.stojmenski@finki.ukim.mk (A.S.); dimitar.trajanov@finki.ukim.mk (D.T.)

**Keywords:** nasopharyngeal carcinoma, magnetic resonance imaging, tumour segmentation, deep learning, reference standard, inter-observer variability, evaluation methodology

## Abstract

**Background/Objectives:** Deep learning segmentation of nasopharyngeal carcinoma is usually reported as one Dice similarity coefficient (DSC) against one reference mask, although multi-sequence MRI is often delineated separately on each sequence. We aimed to quantify how far the choice of reference changes reported performance, and to compare this with the disagreement between radiologists. **Methods:** We analysed a public dataset of 277 patient records with separately delineated T1-weighted, T2-weighted and contrast-enhanced T1-weighted MRI, 30 of whom were annotated by two radiologists. Tumour volumes were compared without registration. Spatial agreement was measured after rigid registration, with the loss from transferring masks between acquisition grids estimated separately. nnU-Net models were scored against every reference, including the second radiologist’s, in 102 held-out patient records. **Results:** Without registration, the sequence effect on tumour volume was 10.9%, against a reader effect of 5.7% (ratio 1.9, 95% CI 1.40 to 2.49). After rigid registration, per-sequence references agreed at a median DSC of 0.783, against 0.898 between radiologists; in the double-annotated patient records, the paired gap narrowed only from 0.129 to 0.083 (95% CI 0.063 to 0.111) when grid transfer was matched. One fixed set of predictions scored 0.726, 0.685 and 0.673, depending only on the reference (median per-patient reference spread 0.075), and switching the reference sequence moved scores 2.7 to 3.6 times as much as switching the radiologist. **Conclusions:** Part of the cross-sequence difference is a difference in what each sequence makes visible rather than annotation error, so a single-reference DSC on multi-sequence data depends on a rarely stated choice. Performance should be reported against every available reference and read against measured inter-reader agreement.

## 1. Introduction

Nasopharyngeal carcinoma arises from the epithelium of the nasopharynx and is particularly prevalent in east and southeast Asia [1], where its aetiology combines Epstein–Barr virus infection with genetic and environmental factors [2]. Radiotherapy is the mainstay of treatment for non-metastatic disease [1], and dose is prescribed to a target that must first be outlined on imaging [3], so the boundary drawn around the primary tumour carries direct clinical consequence. Delineation variability in radiation oncology is large, and protocols, atlases and training reduce it without removing it [4,5].

Magnetic resonance imaging (MRI) is the modality on which the nasopharyngeal primary is assessed and contoured [3,6], and the examination is multi-sequence. T1-weighted (T1WI), T2-weighted (T2WI) and contrast-enhanced T1-weighted (CE-T1WI) images are acquired in one session and read together [7]. The tumour margin is not equally conspicuous on all three because contrast enhancement recruits marginal tissue that is not visible without it, although unenhanced sequences can support segmentation of comparable accuracy at scale [8]. Datasets built from such examinations may annotate each sequence separately rather than once on a co-registered volume, in which case one patient record carries three reference standards for one tumour.

Automated segmentation of the primary tumour is mature, and studies are now separated by small differences in reported accuracy. A meta-analysis of seventeen studies reports a pooled Dice similarity coefficient (DSC) of 0.78 [9], a study across five hospitals reports values near 0.88 [3], and a single-centre study of 1021 patient records reached 0.79 [6]. Progress is measured almost entirely by agreement with a reference standard, so the stability of that reference decides what these numbers mean.

Two assumptions are usually left implicit when a DSC is reported. The first is that the reference standard is a fixed target, so that a higher score reflects a better model. The second is that the distance remaining to a DSC of 1.0 is model error. Neither is safe. Overlap indices respond to properties of the reference annotation itself [10,11], reference labels in medical imaging are noisy rather than exact [12], and measured inter-reader agreement is a more informative point of comparison for a model score than a nominal maximum of 1.0 [13].

For the dataset used here, neither the agreement between readers nor the movement of a score with the reference has been reported. Its release includes independent annotations by two experienced radiologists on a thirty-patient subset but presents their agreement only as figure distributions without numeric values [7], and work built on the dataset reports accuracy against one per-sequence reference at a time [14,15]. We found no study that measures how far a segmentation score moves when only the sequence that supplied the reference is changed. Reporting guidance for artificial intelligence in medical imaging asks authors to state how the reference standard was defined, by whom, and with what inter-rater variability [16], and on multi-sequence data that request cannot be answered without such a measurement.

This study measures how far the per-sequence reference standards for one tumour differ in multi-sequence nasopharyngeal carcinoma MRI, and how far that difference moves a reported segmentation score. We first compare tumour volumes across sequences, which requires no registration, and set the sequence effect against the effect of changing the radiologist for the same patient records. We then compare the references spatially, which requires registration and resampling between acquisition grids, and estimate how much of the observed disagreement those two technical steps can account for. Finally, we train a conventional nnU-Net baseline [17], score fixed sets of its predictions against every available reference, and read the resulting movement against the disagreement between radiologists. Analyses of acquisition hardware, secondary shape descriptors and the prediction of sequence-unstable cases, and an audit of the distributed files, are reported in the Supplementary Materials.

## 2. Related Work

Tumour segmentation on multi-sequence MRI draws on several bodies of work that have developed largely independently of one another. They comprise the methods built for the disease itself, the metrology of how a segmentation is scored, the measured variability of manual delineation together with the responses to it, and the agreement statistics and reporting standards that govern how both are described. Between them they have established a great deal about how a segmentation result should be produced and how it should be judged. Where they have addressed several admissible labels for one image, as when a model is scored against different fusions of the same readers, the alternatives are readings of a single acquisition. What remains open is the case in which the alternatives are drawn on different acquisitions of the same tumour, which is what the multi-sequence protocols used in this disease routinely create.

### 2.1. Segmentation of Nasopharyngeal Carcinoma and Its Reference Standards

Architectures built for multi-sequence input pass the sequences through modality-specific encoders into a shared decoder that emits one mask [18], works in frequency space [14], or attaches a confidence estimate to the output [19,20]. The datasets they are trained on generally settle the reference before evaluation begins. A 200-patient benchmark in this disease pairs pre-aligned computed tomography acquisitions with a single set of masks per patient record [21], and the most widely used multi-sequence tumour benchmark co-registers four sequences and fuses several raters into one mask [22]. Under those conventions the question of which sequence governs the boundary is answered before release and is no longer visible in the data. The dataset used here annotates each sequence separately [7], and published work on it reports each figure against one of those references [14,15].

### 2.2. Validity of Segmentation Metrics and Evaluation Design

Recommendations for validation-metric selection record that metric choice rather than method quality often decides which method appears to win [23], and a companion catalogue of pitfalls records the dependence of the Dice coefficient on structure size and on the properties of the reference annotation [10]. Twenty metrics have been analysed for their response to the boundary complexity, volume and shape of the reference [11]. Challenge rankings shift under small and individually defensible changes to metric, aggregation and test-case handling [24], and reported progress in medical-imaging machine learning has been attributed, in part, to dataset construction and evaluation design [25]. These analyses vary what is computed against a fixed reference. The converse, one fixed computation scored against an equally admissible alternative reference, has not been quantified on a clinical multi-sequence dataset.

### 2.3. Variability of the Reference Standard

Observer-pair overlap has been measured across several structures in computed tomography [26] and reviewed for head and neck target volumes [5], and segmentation performance degrades under boundary-localised label error while tolerating error elsewhere [27]. The usual response to several annotations is fusion, canonically by estimating a hidden true segmentation together with each rater’s performance [28]. Fusion is itself an evaluation choice: one retinal model scores differently against individual graders, a majority vote and an adjudicated consensus [29], and the method used to construct ground truth from several annotators changes the resulting ranking [30]. A parallel line keeps the disagreement, training on raw multi-rater annotations [31], modelling annotator-specific delineation including in this disease [32], publishing inter-observer variability as a challenge benchmark [33], and using it as the reference for model uncertainty [13] and for referring unreliable segmentations [34]. A recent study of uncertainty in head and neck tumour segmentation states that the link between uncertainty and inter-observer variability remains unexplored [35]. Fusing the per-sequence references into a consensus would remove their disagreement from view rather than measure it.

### 2.4. Derived Measurements and Agreement Statistics

Delineation variability propagates into volumetric biomarkers [36], serves as a sanity check for image-biomarker studies [37], and determines which radiomic features survive perturbation in head and neck carcinoma [38], with shape descriptors being the least reproducible family under inter-observer variation [39]. These studies perturb a delineation within one image. When the question is whether two measurements may be substituted for one another, correlation is the wrong statistic. The intraclass correlation rises with between-subject heterogeneity, so a high coefficient in a heterogeneous sample does not license substitution [40], whereas limits in agreement address substitution directly [41]. Stratifying reported performance by a known source of variation is settled practice for centre and vendor [42], but reporting guidance does not yet ask how far a reported score would move under a different admissible reference.

## 3. Materials and Methods

This retrospective analysis of a public dataset has two parts. The first uses the annotations and measurements released with the dataset and requires no trained model. The second trains a conventional segmentation baseline to test whether the effects measured in the first part survive in a reported score. All analyses are at patient level.

### 3.1. Dataset and Annotations

We used the public dataset of primary nasopharyngeal carcinoma MRI with multi-modality segmentation [7], released on Zenodo under CC BY 4.0 as record 13131827 [43]. It contains information about 277 patients with untreated, histopathologically confirmed primary nasopharyngeal carcinoma scanned at one institution in China between July 2013 and January 2022, with 831 axial acquisitions covering T1WI, T2WI and CE-T1WI. According to the released clinical table, the patients were aged 20 to 91 years (mean 50.7, SD 12.9), 203 (73.3%) were men and 265 (95.7%) had non-keratinising carcinoma; the T category was T1 for 15, T2 for 64, T3 for 116 and T4 for 81 patients, with one coded T0, and most were stage III (140) or IVA (93). The data descriptor directs readers to record 10900202, which is restricted; we used the open record. Two diagnostic radiologists, each with more than ten years of experience, delineated the primary tumour slice by slice on every sequence in ITK-SNAP, drawing the inner lesion boundary to limit partial-volume effect [7]. Thirty patient records were annotated independently by both radiologists on all three sequences. The two annotation sets are distributed in different archives, and we verified that they are distinct rather than duplicated. Which thirty patient records these are was fixed by the data providers, and the data descriptor states no selection criterion, so we cannot report how they were chosen. Their identifiers are not consistent with a simple random draw, since twenty of the thirty are numbered 045 or below while the remaining ten run as high as 274; a random draw of thirty from 277 would place about five in that range (hypergeometric $p<10^{-9}$). We therefore compared them with the other 247 patient records based on the two characteristics the released clinical table supports (Supplementary Section S7). No difference in tumour volume was detected on any sequence, with medians of 7.72, 7.07 and 8.11 ${\mathrm{cm}}^{3}$ against 8.69, 8.99 and 9.74 ${\mathrm{cm}}^{3}$ (Mann–Whitney p=0.27, 0.32 and 0.23); with thirty patient records against 247, this is weak evidence of similarity rather than evidence of none, and the subset’s upper quartile is the lower of the two on all three sequences: 12.4 against 18.3 ${\mathrm{cm}}^{3}$ on T1WI. T category was less balanced: 14 of the 30 were T1 or T2 against 65 of the 246 remaining patient records with a recorded category (that is, 47% against 26% (Fisher p=0.031 and odds ratio 2.44)), although the full T1 to T4 distribution did not differ ($\chi^{2}$p=0.12) and the Fisher value is nominal, since it would not survive correction for the five comparisons made here. We therefore do not treat the subset as being representative, and we read the reader effect as if it has been measured in patients whose disease is probably somewhat earlier, and whose largest tumours are less well represented, than that of the cohort as a whole. All archives were checked against their published MD5 checksums.

Acquisition parameters recovered from the DICOM headers of all 831 series show six scanner models from three vendors at 0.95, 1.5 and 3.0 T (Supplementary Section S2), a median in-plane pixel spacing of 0.43 mm, and a median distance between slice centres of 5.5 mm. The sequences of one examination are not acquired on a common grid: T1WI and T2WI occupy different in-plane grids in 27 of the 30 two-reader patient records, usually differing by a factor of two in terms of pixel size, and with slice positions that can differ between sequences.

### 3.2. Data Preparation

The volume analysis uses the per-sequence tumour volumes released with the dataset, which the data providers computed from each three-dimensional segmentation, so it does not depend on any processing of ours. The median volumes we recover, 8.60, 8.67 and 9.42 ${\mathrm{cm}}^{3}$ for T1WI, T2WI and CE-T1WI, reproduce those printed in the data descriptor. The released tables are semicolon-delimited with a decorative first row, and the morphology table has a two-level header, so both require explicit parsing. The metadata file that the data descriptor names as the source of scanner information is absent from the archive, and acquisition parameters were, therefore, taken from the DICOM headers; the recovered per-model patient-record counts reproduce the descriptor’s own table. All spatial analyses and all model training use the DICOM series and the three-dimensional NIfTI masks, whose header geometry we confirmed to match the image series. The separately distributed two-dimensional export pairs’ images were resampled to 256×256 with masks kept at the native matrix, so that 93.0% of image and mask pairs do not correspond when matched by filename; it was not used. Supplementary Section S1 documents the audit, a form of reporting with an established place in medical imaging [44], and the correction for users of those files.

### 3.3. Registration-Independent Volume Analysis

Tumour volume can be compared across sequences without registration because each volume is measured on that sequence’s own mask in its own space. Within-patient variability across the three sequences is summarised as the relative spread: the difference between the largest and smallest volume divided by their mean. Differences between sequences were tested with the Friedman test followed by pairwise Wilcoxon signed-rank tests with Holm correction. Agreement was quantified with the two-way random-effects, absolute-agreement, single-measure intraclass correlation coefficient, ICC(2,1) [40,45], and with Bland–Altman limits of agreement [41] computed on log volume and reported as ratios because volume error is proportional rather than additive.

In the case of the thirty two-reader patient records, we compared the sequence effect with the reader effect for the same patients. The reader effect is the relative volume difference between the two radiologists, $|V_{1}-V_{2}|/\bar{V_{12}}$, averaged over the three sequences, where the denominator $\bar{V_{12}}=\left(V_{1}+V_{2}\right)/2$ is the mean of the two volumes being compared rather than either one of them. The sequence effect is computed in the same pairwise form, as the mean over the three sequence pairs of $|V_{i}-V_{j}|/\bar{V_{ij}}$ with $\bar{V_{ij}}=\left(V_{i}+V_{j}\right)/2$, averaged over the two readers. A range over three values is larger than a difference between two even when nothing differs, so the range-based spread across sequences is reported only for comparability with the 277-patient analysis. The two effects were compared with a Wilcoxon signed-rank test, and the ratio of their medians with a 10,000-sample bootstrap. A linear mixed model based on log volume, with sequence and reader as fixed effects and a random intercept for patient, gave a second estimator.

To examine what the volume differences do to a category derived from volume, patient records were assigned to tertiles of the 277-patient volume distribution and to groups defined by fixed cut-offs at the cohort median of 8.67 ${\mathrm{cm}}^{3}$ and at 15, 20 and 30 ${\mathrm{cm}}^{3}$, and we counted patient records whose group differed between sequences. These bands are an analytical surrogate chosen to expose instability in a derived category. They are not validated treatment or staging thresholds for nasopharyngeal carcinoma.

### 3.4. Spatial Agreement, Registration and Resampling

A voxel-wise comparison between two per-sequence references needs two steps that a comparison between readers on one sequence does not. The acquisitions are registered, and the moving reference is resampled by nearest neighbour onto the fixed acquisition’s grid, whose pixel size and slice positions differ (Section 3.1). The observed cross-sequence discrepancy, therefore, contains three components: sequence-dependent differences in where the boundary was delineated, residual registration error, and the loss of overlap caused by transferring a mask between grids. We refer to the last two together as the registration and resampling contribution and estimate them separately.

Each pair of sequences was registered within session with a rigid transform in SimpleITK [46], maximising Mattes mutual information estimated from a 10% random sample of voxels with a fixed seed, with a regular-step gradient descent optimiser over a three-level multi-resolution pyramid, which was initialised by aligning the geometric centres of the two volumes. The moving reference was transferred with the resulting transform, and agreement was measured with the Dice coefficient against the fixed reference.

Registration quality was checked without reference to any overlap measure. For each registration we recorded how far it moved the tumour centroid from its position in scanner coordinates, and a displacement of more than 20 mm between acquisitions of one session was classed as a failed registration. Results are reported for all registrations and, as a sensitivity analysis, without the failed ones; the same check was applied to the registrations used for model scoring (Section 3.6).

The grid-transfer floor is the Dice that this pipeline returns when both masks describe exactly the same boundary. For each pair we took the fixed reference, re-delineated it on the moving sequence’s grid as the same physical shape, and transferred it back with the exact inverse of the estimated transform, so that registration error is zero by construction. Re-delineation used shape-based interpolation [47]: a signed distance map of the mask, with its zero level midway between voxel centres, was interpolated linearly at the voxel centres of the other grid and thresholded at zero. This models a reader tracing the same boundary on slices placed at the other sequence’s positions and pixel size. A nearest-neighbour round trip, which cannot represent the tumour between slices and therefore captures only the in-plane part of the loss, is reported as a lower bound.

As the one alternative registration, we repeated every comparison with an affine transform, identical to the rigid pipeline in all other settings, and recorded the distance between the tumour centroids produced by the two transfers. An affine registration that placed the tumour more than 10 mm from the rigid solution was classed as diverged. Agreement with the moving reference placed by scanner coordinates alone, without any registration, was recorded as well.

Finally, the comparison with inter-reader agreement was repeated with the grid transfer matched. In the case of the thirty two-reader patient records, one radiologist’s mask on the fixed sequence was re-delineated on the moving sequence’s grid and transferred back exactly as a cross-sequence reference would be, and compared with the other radiologist’s mask on the fixed sequence, in both directions. This resampling-matched inter-reader agreement carries the grid-transfer loss of a cross-sequence comparison without its registration error, so the gap that remains between the two reflects sequence-dependent delineation together with residual registration error.

### 3.5. Segmentation Models

We used nnU-Net [17] (v2 framework, release 2.1) as a conventional self-configuring baseline because the study makes no architectural claim. Training used the DICOM series and the released three-dimensional masks, which preserve the true voxel spacing; the planner selected anisotropic kernels matching the 5.5 mm through-plane and 0.43 mm median in-plane geometry. Three models used the 3D full-resolution configuration, one per sequence, each trained on that sequence’s image and supervised by that sequence’s reference for 250 epochs on a fixed fold. All training masks came from the first radiologist, whose annotations are distributed in the primary archive; the second radiologist’s masks were used for evaluation only and never for training. A fourth model used the 2D configuration on T1WI, and a second training run of the T1WI 3D model, identical except for random initialisation and data order, estimated training variability. Training and validation loss for every configuration, and the validation pseudo-Dice of the two T1WI 3D runs, are given in Supplementary Section S8. A fifth model repeated the T1WI 3D configuration on half the training patient records, 68 of the 135, drawn once with a fixed seed from the training list alone, with the validation and held-out partitions, the preprocessing, the plans, the trainer and the number of epochs unchanged. It supplies a second model that differs from the first in the size of its training set, which Section 4.5 uses to test whether a reported gain survives a change in reference. Each configuration was trained once, so this design does not separate the effect of training-set size from run-to-run training variability.

The split was imposed rather than left to the default, because nnU-Net’s own cross-validation split placed 25 of the 30 two-reader patient records in the training fold. We supplied a fixed patient-level partition, used identically by every model, with all two-reader patient records held out; the evaluation scripts re-verify it before reporting and abort if any evaluated patient record was seen in training. Table 1 gives the patient-record flow and the reason for each exclusion.

### 3.6. Multi-Reference Evaluation of Model Scores

Each model was applied to the held-out patient records. Its predictions were compared with its own sequence’s reference in native space and with the other two references transferred by the rigid registration of Section 3.4, so that the model, its weights and its outputs are identical across comparisons and only the reference changes. For each patient record we define the reference spread as the difference between the best and the worst score that one prediction receives across the three references: $\max_{r}{\mathrm{DSC}}_{r}-\min_{r}{\mathrm{DSC}}_{r}$. The quantity is a range over references and has nothing to do with diagnostic sensitivity; we call it a spread for that reason. It is an observed quantity and includes the registration and resampling contribution for the two transferred references. Where two parts of it are compared, we use its pairwise form, the absolute difference between the scores against two references, so that both sides of a comparison are differences between two values.

Three analyses separate the technical components at model level. The model’s own reference was re-delineated on each other sequence’s grid and transferred back, as in Section 3.4, and the prediction was scored against that copy. The drop from the native score is the grid-transfer loss, and the further difference to the score against the other sequence’s reference is attributable to the reference, together with residual registration error. The T1WI model was also re-scored with the affine registration. For the thirty two-reader patient records, each model was scored against the second radiologist’s mask on every sequence. On the model’s own sequence, this involves no registration, and on the other two the second reader’s mask passes through the same transform as the first reader’s, so a change in reader and a change in sequence can be compared with the registration held fixed. Reader and sequence effects on the score were computed in the pairwise form used for volume.

Segmentation performance is reported with the Dice coefficient and with measures that respond to boundary placement, namely surface Dice at 1 mm and 2 mm tolerance, the 95th percentile Hausdorff distance, the average symmetric surface distance and the signed relative volume error [10,23], which were all computed on the true voxel spacing.

To give the reference spread a practical scale, the 3D and 2D T1WI models were compared against each reference with paired Wilcoxon tests. Two configurations of one framework trained on the same data are expected to perform similarly, so this comparison serves only as a magnitude reference; it is not a test of whether the choice of reference changes model rankings.

### 3.7. Statistical Analysis and Reproducibility

Distributions are summarised by their median and interquartile range (IQR). All tests are two-sided at the 0.05 level. Paired comparisons use the Wilcoxon signed-rank test, comparisons across the three references use the Friedman test with Holm-corrected pairwise follow-up, and 95% confidence intervals (CI) for medians and for ratios of medians use 10,000 bootstrap resamples of patient records. Multiplicity is handled as follows. Each set of pairwise follow-ups to a Friedman test over the three references forms one family and is Holm-corrected within it, as are the three model-against-model comparisons on a neutral reference in Section 4.4; corrected values are the ones reported. The primary quantities of the study are the paired sequence-against-reader contrasts and the per-patient reference spread, which are estimated with bootstrap confidence intervals rather than tested as a family, so no correction applies to them. The remaining comparisons, across measures, models and sequence pairs, are descriptive and are reported without correction, and we draw no conclusion from any of them that rests on a single threshold. The analyses are scripted and deterministic with fixed seeds, every Table and Figure is regenerated by numbered scripts in the accompanying code, and the patient-level split carries a hash that proves the assignment identical across runs. The code that produces every reported number and Figure, together with the fixed split, is available at https://github.com/ema-pandilova/npc-multireference-eval (accessed on 31 October 2026). The software was Python 3.10 with SimpleITK 2.5, SciPy 1.15, statsmodels 0.14 and nnU-Net 2.1 on PyTorch 2.4, and models were trained on a single NVIDIA Quadro RTX 8000 GPU.

## 4. Results

The results are set out in six steps, followed by the reporting items they support: agreement between sequences and between readers without registration, the same comparison in space with the registration and resampling cost separated out, the effect of the reference on a trained model’s reported score, a comparison of models on a reference none of them was trained on, a pair of models that do differ, and the scale set by two configurations of one framework. The Dice values below are medians over patient records or sequence pairs unless stated otherwise, and model scores refer to the 102 held-out patient records with complete data (Table 1) unless a smaller subset is named.

### 4.1. Registration-Independent Agreement Between Sequences and Between Readers

The two radiologists agreed closely on every sequence (Table 2). The intraclass correlation for volume ranged from 0.986 to 0.995 and the median relative volume difference from 4.6% to 5.2%, and the limits of agreement indicate that two experienced readers measuring the same tumour on the same sequence differ by roughly −18% to +16%. We had expected poorer agreement on T2WI, where the lesion boundary is least conspicuous and the grid coarsest; this was not observed.

Between sequences the intraclass correlation was 0.978 (95% CI 0.972 to 0.982) across all three and 0.971 to 0.988 pairwise, which is excellent by convention, but the limits of agreement were 2.0 to 2.4 times as wide on the log scale as between readers (Table 2). A volume measured on T1WI can lie from 25% below to 44% above the same tumour measured on T2WI, and between T2WI and CE-T1WI the interval runs from 40% below to 23% above. The differences were also systematic: CE-T1WI returned volumes of about 12% to 17% larger than the unenhanced sequences and was the largest of the three in 199 of 277 patient records (Friedman $\chi^{2}=171.8$, $p=4.9\times 10^{-38}$; every pairwise Holm-corrected Wilcoxon $p\leq 7.3\times 10^{-4}$). The median within-patient spread was 20.0% (IQR 12.5% to 30.2%) and exceeded 10% in 86% of patient records and 20% in 49%. Tumour volume for these patients spans 0.4 to 84 ${\mathrm{cm}}^{3}$, and because the intraclass correlation is a ratio of between-subject to total variance, its high value shows that the sequences rank patient records consistently, not that a volume from one may be substituted for a volume from another in an individual patient record.

**Figure 1:**
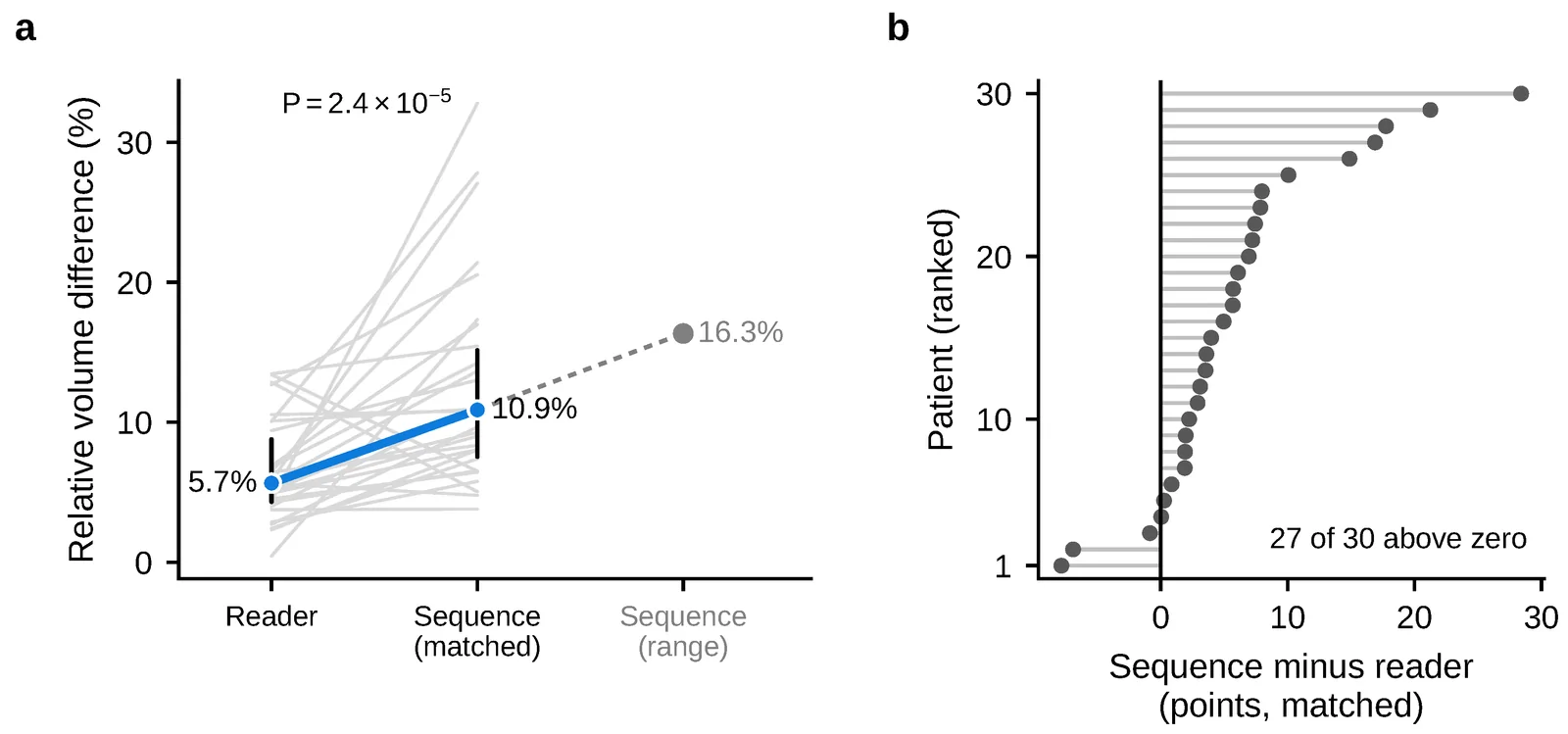
Sequence against reader effect on tumour volume, paired within patient (n=30), without registration. Blue, the matched pairwise estimator reported in the text; and grey, the range-based estimator, shown for comparability only. (**a**) Solid grey lines, individual patients; dashed grey line, the link to the range-based estimator; and bars, interquartile range. (**b**) Per-patient difference, ranked.

For the thirty patients whose information was annotated by both radiologists, the two effects can be compared directly, and still without registration (Figure 1). The sequence effect had a median of 10.9% (IQR 7.6% to 15.1%) against a reader effect of 5.7% (IQR 4.3% to 8.8%), a ratio of 1.9 (bootstrap 95% CI 1.40 to 2.49; Wilcoxon $p=2.4\times 10^{-5}$), and it was the larger of the two in the case of 27 of the 30 patients. The range-based sequence effect, kept for comparability with the 277-patient spread, was 16.3%. The mixed model gave the same ordering. Between-patient differences accounted for 98.0% of the variance in log volume, the systematic sequence shift for 0.58% and the systematic reader offset for 0.017%; T1WI and T2WI volumes were 0.905 and 0.871 times the CE-T1WI volume ($p=9.3\times 10^{-10}$ and $p=2.2\times 10^{-17}$), whereas the reader term was not significant (p=0.14). These shares describe only the constant part of each factor, so the paired effects are the primary estimate.

#### Downstream Consequences for Patient Stratification

The volume differences carry into categories derived from volume. With patient records assigned to tertiles of the 277-patient distribution, 55 of 277 (19.9%) fell into a different tertile depending on which sequence was measured, although none moved between the lowest and the highest; the mean Cohen’s kappa between sequences was 0.80. Fixed cut-offs moved fewer patient records: 22 (7.9%) at the cohort median of 8.67 ${\mathrm{cm}}^{3}$, 17 (6.1%) at 15 and at 20 ${\mathrm{cm}}^{3}$, and 11 (4.0%) at 30 ${\mathrm{cm}}^{3}$. Tertiles of maximum diameter, the descriptor least affected by sequence (Supplementary Section S4), reassigned 64 patient records (23.1%) because its narrower range across patient records places the band boundaries closer together. These bands are an analytical surrogate, so the counts show that a category derived from volume is unstable to the choice of sequence. They do not show that the corresponding proportion of patient records would be treated differently.

### 4.2. Spatial Agreement and the Contribution of Registration and Resampling

**Figure 2:**
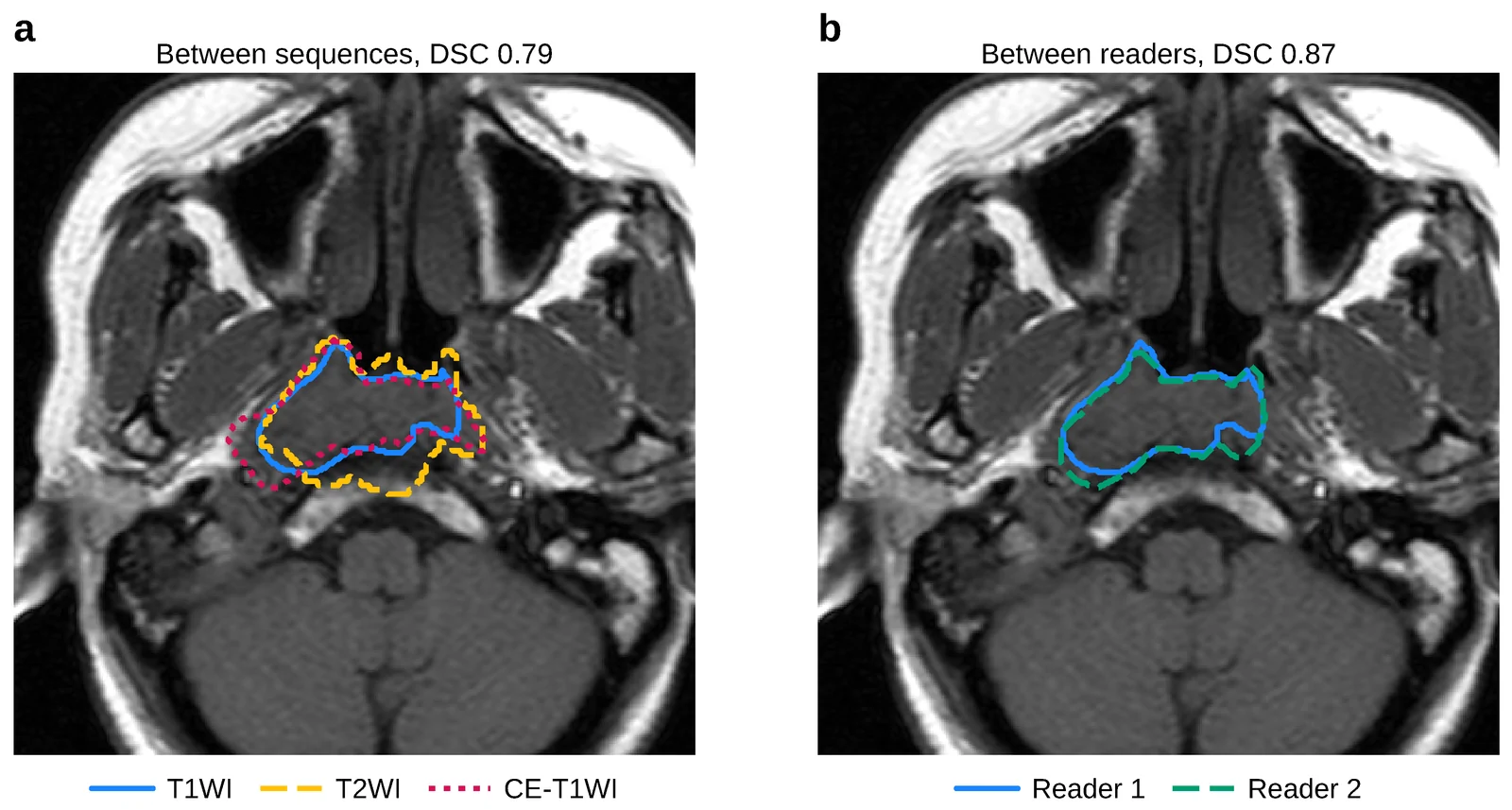
The two disagreements for one patient and one slice. (**a**) The three per-sequence references, with T2WI and CE-T1WI registered onto T1WI. (**b**) The two radiologists on T1WI, with no registration involved; the blue contour is the same annotation in both panels. The case was selected by rule, as the two-reader patient record closest to both dataset medians.

Registered and transferred, the per-sequence references of one tumour agreed at a median Dice of 0.783, with the highest being between the two unenhanced sequences (0.806) and lowest being between CE-T1WI and T2WI (0.759), the pair that differs most in contrast mechanism and in acquisition grid (Table 3). Inter-reader agreement on a single sequence, which needs neither registration nor resampling, was 0.891 to 0.904. Restricted to the thirty two-reader patient records and paired within patient, cross-sequence agreement was 0.770 and inter-reader agreement 0.898, a difference of 0.129 (bootstrap 95% CI 0.096 to 0.155; Wilcoxon $p=3.7\times 10^{-9}$), with inter-reader agreement the higher for 29 of the 30 patients. Figure 2 shows both comparisons for one patient.

Five of the 827 rigid registrations, in three patient records, converged to a wrong alignment. They moved the tumour 22.6 to 130.8 mm from its scanner-coordinate position and reduced the overlap to at most 0.003, where scanner coordinates alone gave 0.628 to 0.865, whereas no other registration moved the tumour by more than 16.9 mm. None of the three patient records was held out for model evaluation or annotated by both radiologists. Excluding the five pairs changes the median agreement from 0.783 to 0.784, and the pair medians to 0.806 for T1WI and T2WI, 0.774 for CE-T1WI and T1WI and 0.766 for CE-T1WI and T2WI.

Part of the discrepancy is the grid transfer itself. A reference re-delineated on the other sequence’s grid and transferred back with the exact inverse transform agreed with itself at a median Dice of 0.955 (IQR 0.889 to 0.983). Moving a mask between these grids, therefore, costs about 0.045 Dice when there is no disagreement at all, against an observed loss of 0.217 between the per-sequence references. The cost was concentrated in pairs acquired at different slice positions: where the two sequences shared slice planes (731 pairs) the floor was 0.961, and where the planes were offset or differently spaced (96 pairs, nearly all involving CE-T1WI), it was 0.888. A nearest-neighbour round trip, which captures only the change in pixel size within a slice, lost almost nothing (median 0.998).

The registration itself mattered little. The rigid registration moved the tumour by a median of 1.3 mm from its scanner-coordinate position (IQR 0.7 to 2.5 mm), and without any registration, with the moving reference placed by scanner coordinates alone, the median agreement was 0.772. The affine registration converged in 738 of the 827 pairs. In the other 89 (10.8%), 85 of them with CE-T1WI as the fixed image, its additional degrees of freedom let the optimiser move the tumour more than 10 mm from the rigid solution. Where it converged, it changed cross-sequence agreement by a median of +0.002 (from 0.787 to 0.791), and the rigid and affine transfers of the same mask agreed at a median Dice of 0.968 with tumour centroids 0.47 mm apart.

With the grid transfer matched, the gap between the radiologists narrowed but did not close. When one radiologist’s mask was passed through the other sequence’s grid in the same way as a cross-sequence reference, inter-reader agreement fell from 0.898 to 0.861, while cross-sequence agreement for the same thirty patients, averaged over both radiologists, was 0.764. The median per-patient gap fell from 0.129 to 0.083 (bootstrap 95% CI 0.063 to 0.111; Wilcoxon $p=2.6\times 10^{-8}$), and the matched inter-reader agreement remained the higher for 27 of the 30 patients. This remainder contains the sequence-dependent delineation differences together with any residual registration error, which these data cannot separate.

### 4.3. Effect of the Reference on Reported Segmentation Scores

**Figure 3:**
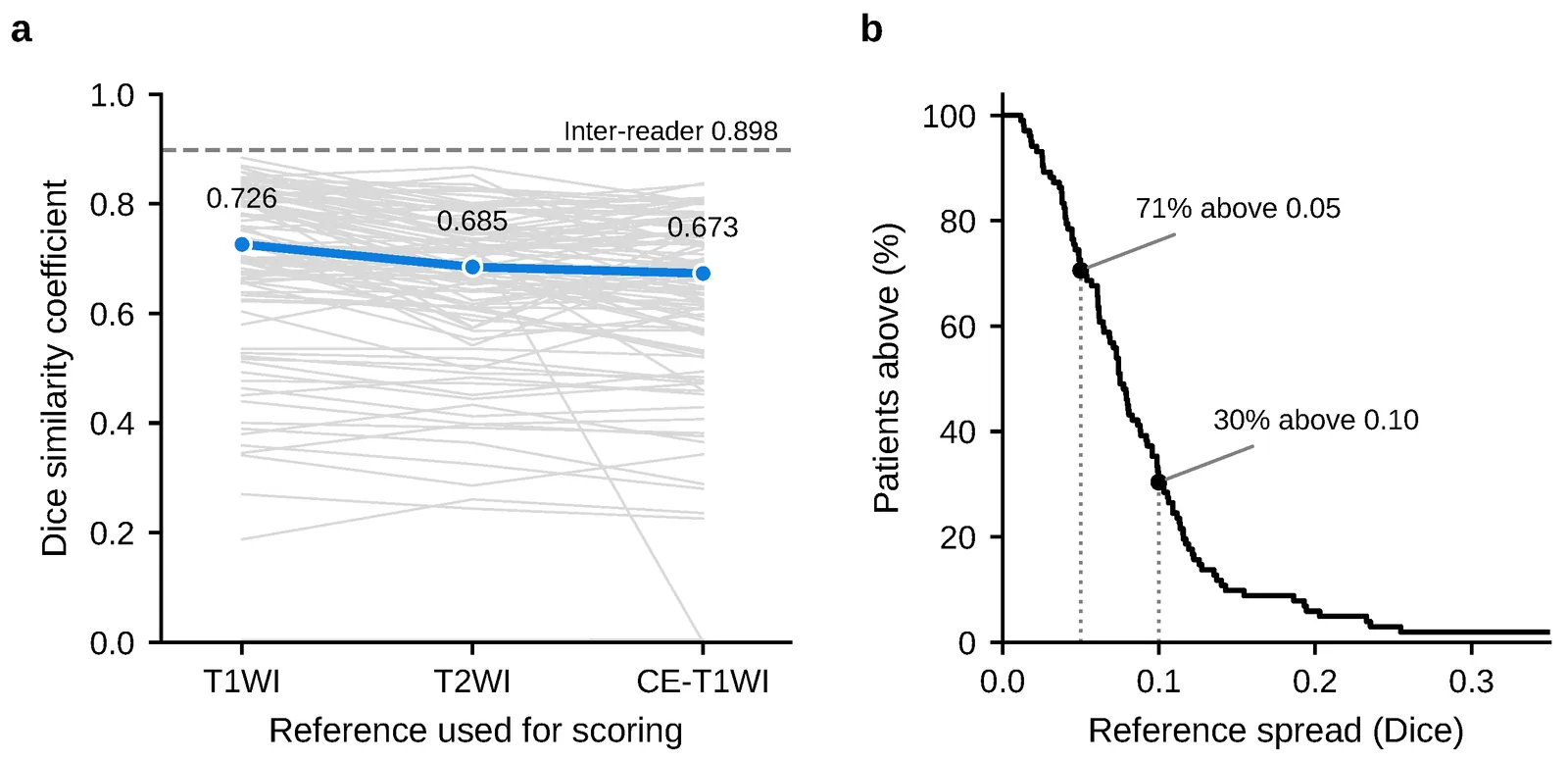
One fixed set of predictions from the T1WI 3D model scored against each reference. (**a**) Every held-out patient record with complete data, one line each; the model is unchanged between columns. (**b**) Per-patient reference spread; one patient record lies beyond the axis range.

The T1WI 3D model was scored against every reference in the 102 held-out patient records with complete data (Table 1). Median Dice was 0.726 against T1WI (IQR 0.643 to 0.810), 0.685 against T2WI (0.590 to 0.747) and 0.673 against CE-T1WI (0.563 to 0.754), with the model, its weights and its outputs unchanged between the three (Figure 3). The differences were systematic (Friedman $\chi^{2}=63.1$, $p=2.0\times 10^{-14}$); Holm-corrected Wilcoxon tests separated T1WI from both other references ($p=2.2\times 10^{-9}$ and $p=1.5\times 10^{-14}$) but not T2WI from CE-T1WI (p=0.058). The median per-patient reference spread was 0.075 (bootstrap 95% CI 0.065 to 0.087); it exceeded 0.05 for 71% of patients and 0.10 for 30%. Boundary-sensitive measures moved further than Dice (Table 4). Surface Dice at 1 mm had a median reference spread of 0.107, the average symmetric surface distance moved by a median of 0.61 mm, and the relative volume error by 16.2 percentage points.

The technical components account for little of this at model level (Supplementary Section S6). When a model’s own reference was passed through another sequence’s grid and transferred back, its score changed by a median of at most 0.004, although it fell by more than 0.02 for 11% to 33% of patients, mostly where CE-T1WI was involved. Scored against two references that carry the same grid transfer, the own reference passed through the other grid and the other sequence’s reference, the models still scored lower against the other sequence (by a median of 0.026 to 0.048 across the six combinations of model and sequence), and every confidence interval excluded zero; without this matching, the drop was 0.030 to 0.056. Quality control flagged three of the 612 registrations used for model scoring, all transferring a CE-T1WI reference: patient record 169 for the T1WI and T2WI models and patient record 234 for the T2WI model. Each moved the tumour 84 to 246 mm and reduced the score against that reference to zero, where scanner coordinates alone gave 0.576 to 0.679, while no other registration moved it by more than 8.9 mm. Excluding the affected patient records, the T1WI model’s median reference spread was 0.074 (95% CI 0.062 to 0.087), and across the three models it ranged from 0.071 to 0.074. For the same 101 patients, the affine registration gave the T1WI model a median reference spread of 0.072 (95% CI 0.060 to 0.082), against 0.074 with the rigid one.

All three trained models showed the same pattern (Table 5). The reference spread was 0.075, 0.071 and 0.072 with T1WI, T2WI and CE-T1WI as the evaluation space, and each model scored highest against its own reference, which it was trained on and which alone is compared without registration. The pairwise form separates that advantage from the rest. The median absolute difference between a model’s own reference and a transferred one was 0.046 to 0.057, and between the two transferred references, neither of which the model was trained on, it was 0.033 to 0.040, which is 58% to 78% as large. Most of the reference spread, therefore, remains when the advantage of the model’s own reference is removed, and it is as stable across evaluation spaces as the full quantity.

The second radiologist’s masks put these movements on a human scale (Table 6). In the thirty two-reader patient records, switching the reference from one radiologist to the other on the same sequence changed a model’s Dice by a median of 0.012 to 0.016, whereas switching the sequence changed it by 0.042 to 0.050, which is between 2.7 and 3.6 times as much. The sequence effect was the larger for 27 of the 30 patients from each of the three models. Restricted to the two references transferred into the model’s space, which removes the advantage of its own reference, the sequence effect remained 1.8 to 2.2 times the reader effect, and every confidence interval excluded one. In this restricted comparison, both radiologists’ masks pass through the same transform whereas the two sequences use different ones, so part of the remaining sequence effect may still be registration error.

**Figure 4:**
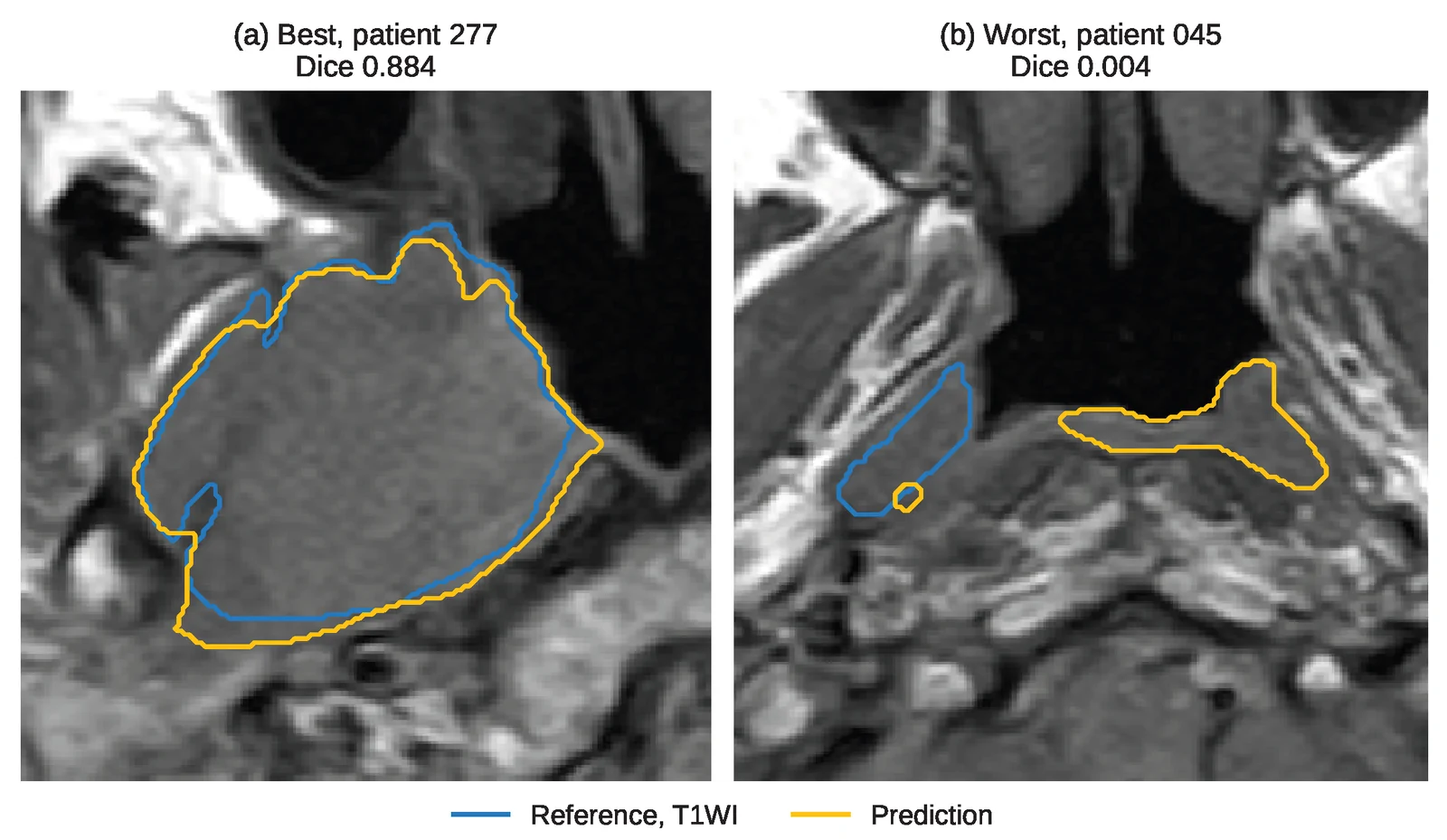
The best and the worst held-out case for the T1WI 3D model, scored against the T1WI reference in native space, which involves no registration. Blue, the reference; and yellow, the prediction. Both cases were selected by rule as the extremes of the per-patient Dice distribution, and the slice shown is the one carrying the largest reference area. (**a**) Patient 277, Dice 0.884, tumour volume 26.4 ${\mathrm{cm}}^{3}$. (**b**) Patient 045, Dice 0.004, tumour volume 1.04 ${\mathrm{cm}}^{3}$.

Where the model fails is visible at the two ends of the distribution (Figure 4). The best held-out case was patient 277, a 26.4 ${\mathrm{cm}}^{3}$ tumour whose boundary the model followed closely at a Dice of 0.884, with the two contours separating only along the inferior margin. The worst was patient 045, with a 1.04 ${\mathrm{cm}}^{3}$ lesion of low conspicuity, where the model placed most of its output away from the reference and scored 0.004. The pattern behind these two cases holds across the held-out set: the score rose with tumour size (Spearman ρ=0.31, p=0.002), with a median Dice of 0.675 in the smallest quartile, at most 5.00 ${\mathrm{cm}}^{3}$, against 0.764 in the largest, at least 16.69 ${\mathrm{cm}}^{3}$. The three patients whose information scored below 0.30 had a median tumour volume of 2.71 ${\mathrm{cm}}^{3}$ against 8.61 ${\mathrm{cm}}^{3}$ for the cohort. Small tumours are, therefore, where the model is least reliable, and also where a change in reference moves the score most, since the same absolute boundary shift is a larger fraction of a small volume. Size is the association the held-out set supports; the low conspicuity of the worst case is an observation about that image and was not tested as a cause across the cohort.

### 4.4. Comparing Models on a Reference Neither Was Trained on

The matrix in Table 5 reorders the three models when the reference changes, but it cannot be read as evidence about ranking because each model is scored against the reference it was trained on and that reference alone is compared without registration. For any pair of the three there is exactly one reference that neither was trained on, and scored against it the two are on equal terms, since both predictions meet a reference foreign to both and both carry the same registration and grid transfer.

Under that constraint the apparent reordering disappears (Table 7). Every pair changed winner across the three references, and in each case the change was produced by whichever reference favoured one member: the paired difference ran from +0.034 to +0.084 in favour of the model whose own reference was in use, and reversed when the other member’s reference was used. On the neutral reference none of the three pairs separated. The paired differences were +0.004, +0.022 and +0.019, every bootstrap interval included zero, and the Holm-corrected signed-rank values were 0.97, 0.14 and 0.19.

These three models, therefore, cannot test whether a ranking survives a change in reference because on a neutral footing no statistically distinguishable ranking among them was established. What the Table does establish is narrower. The reordering in Table 5 is consistent with an own-reference advantage, which accounts for what is seen without any need to invoke a reversal of ranking by the reference. That mechanism is not established as the only explanation: these comparisons are consistent with it, but they cannot exclude a further contribution from the reference itself that is too small for this design to resolve. Nor does the Table establish that the three models are alike: the intervals are wide, and two of them admit differences as large as 0.049 and 0.031 Dice, either of which would exceed the gain that separates the two models of Section 4.5. Failing to separate here is a limit on what these three models can resolve, not evidence of equivalence, and a study wanting to claim that reference choice reverses rankings needs models separated by more than these are.

### 4.5. A Model Pair Separated by Training-Set Size

Section 4.4 leaves the question of rankings open, because the three per-sequence models cannot be separated on a neutral footing. Testing whether a reported gain survives a change in reference needs two models that do differ. We produced one such pair by training the T1WI 3D configuration a second time on half the training patient records: 68 of the 135, drawn once with a fixed seed. The validation and held-out partitions, the preprocessing, the plans, the trainer and the number of epochs were unchanged, so the size of the training set is the only deliberate difference between the two.

Differences below are per patient record and in the direction full minus reduced, so a positive value means the full model scored higher. The reduced model scored lower, and the difference kept that sign against all three references (Table 8). The full model led by a median of 0.022, 0.018 and 0.026 Dice per patient record against the T1WI, T2WI and CE-T1WI references, every bootstrap interval excluded zero, every Holm-corrected signed-rank test was significant at $p<10^{-4}$, and the matched-pairs rank-biserial correlation ran from 0.45 to 0.53. A gain of this size, therefore, survives a change in reference intact, which the comparison between the 3D and 2D configurations could not establish, since no significant difference between those two was detected.

Two things qualify that. The size of the gain depends on which reference is used to measure it, running from 0.018 to 0.026, so the reference changes what a paper would report as the benefit of the extra data even where it does not change the conclusion. And the gain is small beside the reference spread: the full model’s median spread of 0.075 is about three times the largest of the three gains, so for one fixed model the choice of reference still moves the score further than halving its training set does. The reduced model carried a median reference spread of 0.074 (95% CI 0.066 to 0.083), against 0.075 for the full model, so the weaker model inherited the spread essentially unchanged. That is what would be expected if the spread is driven by the references rather than by model quality, though two models of one architecture cannot establish it.

Two further limits apply. This is one of reduced training size, not a training-size gradient, and it speaks to a gain between two models of the same architecture; whether the choice of reference can reverse the ranking of models that differ in architecture remains untested here. And each configuration was trained once, so the comparison does not separate the size of the training set from run-to-run training variability: retraining the full configuration with a different initialisation and data order moved its median Dice by 0.006 to 0.021 (Section 4.6), a range that overlaps these differences, so the magnitude of the gain should not be read as the effect of data volume alone. What the paired tests establish is narrower and is unaffected by this: the full model scored higher for the great majority of patient records against every reference, and that ordering did not depend on which reference was used.

### 4.6. Scale Comparison with Two nnU-Net Configurations

Median Dice for the 3D and 2D configurations of the T1WI model was 0.726 and 0.722 against the T1WI reference, 0.685 and 0.684 against T2WI, and 0.673 and 0.676 against CE-T1WI. The differences between the medians, computed before rounding, were +0.0038, +0.0012 and −0.0029 (Wilcoxon p=0.36, p>0.99 and p=0.87). No statistically significant difference between the two configurations was detected against any reference, and the per-patient difference between them did not depend on the reference (Friedman $\chi^{2}=4.79$, p=0.091). Retraining the 3D configuration with a different random initialisation and data order moved its median Dice by 0.006, 0.021 and 0.012 against the three references, again without a significant paired difference (Wilcoxon p=0.95, p=0.81 and p=0.35), and for each patient record, the two runs differed by a median of 0.015 to 0.020. The difference between the configurations, therefore, lies within the variation produced by retraining one of them.

For the same 3D predictions, the median moved by 0.053 between the T1WI and the CE-T1WI reference, which is about fourteen times the largest difference between the configurations and two and a half times the largest change produced by retraining. The figures are of different kinds: a shift in one model’s median across references against differences between models or training runs scored against one reference, and we do not test one against the other. The comparison shows only that a movement produced by the reference alone can be larger than the differences separating closely performing configurations, and larger than the variation from retraining one of them. Because no significant difference between these configurations was detected, it says nothing about whether the choice of reference changes the ranking of models that do differ.

### 4.7. Multi-Reference Reporting Recommendations

The measurements above translate into eight reporting items for datasets that provide more than one admissible reference standard (Table 9). Each item can be checked from a manuscript alone. Items 1 and 2 describe the references, items 3 to 5 report performance against them, item 6 supplies the human scale, item 7 makes the boundary disagreement visible, and item 8 exposes how much of a reported difference the evaluation pipeline itself introduces.

## 5. Discussion

The per-sequence reference standards for one nasopharyngeal carcinoma differed more than two radiologists did on one sequence. This held for tumour volume, which needs no registration, where the sequence effect was about twice the reader effect and the larger of the two for 27 of 30 patients, and it held spatially after registration. The difference carried into a trained model’s reported score. One fixed set of predictions scored 0.726, 0.685 or 0.673, depending only on which reference was used, which is a median per-patient reference spread of 0.075. For the patient records annotated twice, switching the sequence of the reference moved a model’s score 2.7 to 3.6 times as much as switching the radiologist.

### 5.1. What the Cross-Sequence Discrepancy Contains

The three per-sequence masks are not three attempts at one fixed boundary. Contrast enhancement makes marginal tissue conspicuous that is not visible without it, so a radiologist drawing the lesion on CE-T1WI is in part delineating something different from the same radiologist drawing it on T1WI, and the difference between the two is a difference in the target rather than an error by either. That is why we treat the three as alternative references rather than as one reference and two deviations from it, and it is the reason the choice has to be reported rather than reconciled. What follows bounds how much of the measured difference is technical.

A spatial comparison between sequences cannot be made without registration and resampling, and both lower the measured agreement whatever the true relation between the references. The analyses here bound that technical part from three directions. Tumour volume needs neither step, and the volume comparison still shows a sequence effect of about twice the reader effect, so the phenomenon does not originate in the registration. Transferring a mask between these grids costs about 0.045 Dice even when two masks describe the same boundary, a fifth of the observed loss, and most of that cost falls on sequences acquired at different slice positions. With the transfer matched, the gap to inter-reader agreement fell from 0.129 to 0.083 but did not close. The transform class changed agreement by 0.002 where the affine optimisation converged, and the few rigid registrations that failed outright moved the median by 0.001. What remains after these adjustments is sequence-dependent delineation together with any registration error that the rigid and affine transforms share, and these data cannot divide it further. The spatial figures are, therefore, best read as an upper bound on the delineation component, and the volume comparison as registration-free evidence that the component exists.

The direction of the systematic part is consistent with the imaging physics. CE-T1WI returned the largest volume in 199 of 277 patient cases and about 12% to 17% more than the unenhanced sequences, which is as expected if enhancement recruits marginal tissue that is not conspicuous without contrast. Unenhanced sequences can, nonetheless, support segmentation of comparable accuracy at scale [8], so which sequence should govern the boundary is a question of definition and reporting rather than of capability.

### 5.2. Interpretation of the Agreement Statistics

The intraclass correlation between per-sequence volumes was 0.978, which is excellent by convention, while the limits of agreement admit differences ranging from 40% below to 44% above. The two statistics answer different questions. The intraclass correlation is a variance ratio that grows with the heterogeneity of the sample, and tumour volume here spans two orders of magnitude, so it reports that the references rank patient records similarly, which they do. The limits of agreement report whether one may be substituted for another for an individual patient, and they show that it may not. The same caution applies to model scores: a high Dice against one reference does not show that a model agrees with any other reference, and Section 4.3 shows directly that it does not.

### 5.3. Relation to Previous Work

Inter-reader variability as a reference for model uncertainty [13], centre- and vendor-stratified evaluation [42], multi-rater modelling including in this disease [32], and the propagation of delineation variability into derived biomarkers [36,38] are established. We did not find work that sets those sources against the choice of reference sequence itself. In these data the sequence effect on volume was about twice the reader effect on a matched estimator, and in the mixed model its systematic component was the larger fixed effect by more than an order of magnitude, while the reader term was not significant. Work on this dataset tabulates accuracy per sequence [14] or draws radiomic features from each sequence’s region in turn [15], and none of it quantifies how far a score moves when only the reference sequence changes.

### 5.4. Implications for Reporting and Generalisability

The remedy does not require new methods, only that the reference be treated as an experimental variable, as the items in Table 9 set out. When a reported difference between models is smaller than the reference spread of the dataset, its robustness to the choice of reference should be shown against every available reference rather than assumed. A paired comparison against one reference can still detect a real difference between models; what it cannot show on its own is that the difference survives a change in reference. Results from studies that made different reference choices should not be compared directly unless both report the choice.

The magnitudes measured here are specific to this dataset, and we would not expect the same reference spread elsewhere. The mechanism is more general. Tumour boundaries are not equally conspicuous across sequences, so wherever a dataset annotates each sequence separately, the references will disagree to some degree, and any model scored against one of them inherits that disagreement. Multi-parametric prostate MRI, multi-phase liver imaging, breast dynamic-contrast-enhanced MRI and multi-sequence brain-tumour protocols share this structure. Datasets that co-register the sequences and release a single mask do not show the disagreement because the choice of sequence was made before release; their scores are equally conditional on that choice but offer no way to measure it. In that sense, the dataset used here is unusually informative rather than unusually flawed.

### 5.5. Limitations

The 277 patients come from one institution, and the reader comparison rests on the thirty patient records annotated by both radiologists, who were two readers from the same centre. How those thirty were chosen is not documented, and no difference in their tumour volumes was detected, although those showing the largest tumours are less well represented, and they carry a higher proportion of T1 and T2 disease, so the reader effect is measured in terms of patients with a somewhat earlier stage of disease than the sequence effect is. We could not replicate the analysis externally because we are not aware of a second public dataset that annotates each sequence separately. The grid-transfer floor relies on shape-based interpolation between slices 5.5 mm apart, which models how a reader would trace the same boundary on the other grid; a real re-delineation could differ, and the nearest-neighbour round trip gives the lower end of the loss. Registration error shared by the rigid and affine transforms is not bounded by comparing them, so the matched spatial gap combines sequence-dependent delineation with such error, and the volume comparison is the evidence that does not depend on it. The rigid registration was initialised by aligning the geometric centres of the two volumes, which, for sequences covering different fields of view, can start the optimiser far from the true alignment. Quality control found five failures among the 827 registrations between references and three among the 612 used for model scoring, and every conclusion holds with and without them; initialising from scanner coordinates would avoid most such failures, and we would recommend it. Each model was trained on one fixed fold, and only the T1WI 3D configuration was trained twice, so the training variability of the other models was not estimated; for the configuration that was, retraining moved the median Dice by up to 0.021. No significant difference between the two nnU-Net configurations was detected, which places a loose bound on how far apart they are without establishing that they perform alike, so their comparison supplies a scale only. The three per-sequence models cannot fill the gap either: on the one reference that neither member of a pair was trained on, no pair separated (Section 4.4), which is consistent with the reordering in Table 5 arising from the own-reference advantage rather than from the reference choice, although it neither establishes that advantage as the sole cause nor establishes any statistically distinguishable ranking among the three models, which are, equally, not shown to be equivalent. The reduced-training-set pair of Section 4.5 does differ, and its gain held its sign and its significance against all three references, so a gain of that size survives the choice. That is one pair at one reduced training size and of one architecture, and it shows only that such a gain can survive, not that every gain does. Whether the choice of reference can reverse the ranking of models separated by architecture rather than by training data is still untested, and would need models that differ in that way. Finally, the stratification bands are an analytical surrogate rather than validated clinical thresholds, and because the dataset records progression-free survival without an event indicator, no outcome analysis was possible.

## 6. Conclusions

On a public 277-patient multi-sequence nasopharyngeal carcinoma dataset, the per-sequence reference standards for the same tumour differed more than two radiologists did on one sequence (in terms of tumour volume without registration and spatially after registration), and registration and resampling accounted for only part of the spatial difference. A trained nnU-Net inherited the difference. One fixed set of predictions scored between 0.726 and 0.673, depending only on the reference, a median per-patient reference spread of 0.075, and changing the sequence of the reference moved a model’s score 2.7 to 3.6 times as much as changing the radiologist.

A single-reference Dice on multi-sequence data is, therefore, conditional on a choice that is rarely stated. We recommend that studies report performance against every available reference, publish the per-patient reference spread, state their registration and resampling and report what those steps cost, and read their scores against measured inter-reader agreement rather than against a nominal maximum of 1.0.

## Figures and Tables

**Table 1:** Patient-record flow and reasons for exclusion.

Stage	Basis	Patients
Released dataset	T1WI, T2WI and CE-T1WI, each annotated	277
Excluded, patient 159	T1WI slice spacing 5.66 mm against 5.5 mm in the mask	−1
Excluded, patient 188	T1WI image 38 slices against 31 in the mask	−1
Entering the model pipeline		275
Training	fixed patient-level split	135
Validation	fixed patient-level split	37
Held out	withheld from training and validation	103
Excluded, patient 259	T2WI image 32 slices against 18 in the mask, so that reference could not be transferred	−1
Scored against all three references	complete data	102

**Table 2:** Tumour volume agreement without registration: two radiologists on the same sequence (n=30) and two sequences for the same radiologist (n=277). Bias and limits of agreement are log-scale ratios.

Comparison	ICC(2,1)	Bias (Ratio)	Limits of Agreement	Median |ΔV| (%)
Reader 1 against reader 2, same sequence				
T1WI	0.995	0.97	0.82–1.13	5.01
T2WI	0.986	0.99	0.84–1.16	5.20
CE-T1WI	0.989	0.99	0.85–1.14	4.59
One sequence against another, same reader				
T1WI vs. T2WI	0.988	1.04	0.75–1.44	9.54
T1WI vs. CE-T1WI	0.976	0.89	0.63–1.26	11.95
T2WI vs. CE-T1WI	0.971	0.86	0.60–1.23	15.24
All three	0.978			

**Table 3:** Spatial agreement between per-sequence references (median Dice). Floor, a reference compared with its own copy passed through the other grid and back; and NN, the same with nearest-neighbour resampling. Affine values are for the 738 pairs where it converged.

Comparison	*n*	Dice	Dice, Affine	Grid-Transfer Floor	NN Round Trip
Two radiologists, same sequence; no registration or resampling					
T1WI	30	0.891	–	–	–
T2WI	30	0.904	–	–	–
CE-T1WI	30	0.901	–	–	–
One radiologist, two sequences; registered and transferred					
T1WI vs. T2WI	275	0.806	0.803	0.979	0.995
CE-T1WI vs. T1WI	276	0.773	0.778	0.931	1.000
CE-T1WI vs. T2WI	276	0.759	0.781	0.933	0.998
All pairs	827	0.783	0.791	0.955	0.998
Thirty two-reader patients, per-patient medians					
Cross-sequence, both radiologists	30	0.764	–	–	–
Inter-reader, native	30	0.898	–	–	–
Inter-reader, grid transfer matched	30	0.861	–	–	–

**Table 4:** Scores for the T1WI 3D model against each reference (102 patients, medians). Reference spread is the per-patient best-minus-worst value. HD95, 95th percentile Hausdorff distance; ASSD, average symmetric surface distance; and RVE, signed relative volume error.

Measure	T1WI	T2WI	CE-T1WI	Reference Spread
Dice	0.726	0.685	0.673	0.075
Surface Dice, 1 mm	0.696	0.626	0.604	0.107
Surface Dice, 2 mm	0.778	0.732	0.719	0.092
HD95 (mm)	6.12	6.28	6.62	0.98
ASSD (mm)	1.38	1.56	1.87	0.61
RVE (%)	−5.5	−5.3	−16.1	16.2

**Table 5:** Median Dice of each 3D model against each reference (102 patients); bold marks the training reference. Reference spread is given as the range over three references and in pairwise form. The last column compares two scores that share the same grid transfer (Supplementary Section S6).

	Reference Used for Scoring			Reference Spread			
Model Trained on	T1WI	T2WI	CE-T1WI	Range	Own vs. Transferred	Transferred Pair	Beyond Grid Transfer
T1WI	**0.726**	0.685	0.673	0.075	0.051	0.040	0.036 to 0.038
T2WI	0.702	**0.723**	0.679	0.071	0.046	0.034	0.026 to 0.035
CE-T1WI	0.693	0.669	**0.744**	0.072	0.057	0.033	0.030 to 0.048
Median				0.072	0.051	0.034	

**Table 6:** Median per-patient change in a model’s Dice when the reference is switched between radiologists or between sequences (30 patients). Ratio, sequence effect over reader effect, with 95% CI.

		All References			Transferred References Only		
Model Trained on	*n*	Reader	Sequence	Ratio	Reader	Sequence	Ratio
T1WI	30	0.012	0.042	3.4 (2.1 to 5.2)	0.015	0.033	2.2 (1.4 to 3.4)
T2WI	30	0.016	0.043	2.7 (2.0 to 4.0)	0.016	0.029	1.8 (1.3 to 3.0)
CE-T1WI	30	0.014	0.050	3.6 (2.4 to 7.6)	0.013	0.024	1.9 (1.2 to 3.3)

**Table 7:** Each pair of models compared under each reference (102 patients). Median of the per-patient Dice difference, first model minus second, with a 10,000-sample bootstrap interval. Neutral marks the reference that neither model in the pair was trained on; the other two rows of a pair each favour one member.

Pair	Reference	Status	Median Difference	95% CI
T1WI vs. T2WI	T1WI	favours T1WI	+0.036	+0.021 to +0.052
	T2WI	favours T2WI	−0.044	−0.059 to −0.029
	CE-T1WI	neutral	+0.004	−0.020 to +0.021
T1WI vs. CE-T1WI	T1WI	favours T1WI	+0.034	+0.020 to +0.072
	T2WI	neutral	+0.022	−0.012 to +0.049
	CE-T1WI	favours CE-T1WI	−0.068	−0.082 to −0.051
T2WI vs. CE-T1WI	T1WI	neutral	+0.019	−0.004 to +0.031
	T2WI	favours T2WI	+0.084	+0.048 to +0.113
	CE-T1WI	favours CE-T1WI	−0.061	−0.082 to −0.041

**Table 8:** The full T1WI 3D model against the same configuration trained on half the training patient records (102 held-out patient records). Median of the per-patient Dice difference, full minus reduced, with a 10,000-sample bootstrap interval, Holm-corrected signed-rank *p* over the three references, and the matched-pairs rank-biserial correlation.

Reference	Full (135)	Reduced (68)	Difference	95% CI	Holm *p*	*r*
T1WI	0.726	0.680	+0.022	+0.010 to +0.039	$1.7\times 10^{-5}$	0.51
T2WI	0.685	0.672	+0.018	+0.007 to +0.038	$6.9\times 10^{-5}$	0.45
CE-T1WI	0.673	0.644	+0.026	+0.007 to +0.039	$9.4\times 10^{-6}$	0.53

**Table 9:** Reporting items for datasets with more than one admissible reference standard, and where each is addressed in this study.

	Item	Here
1	State which sequence or image supplied each reference standard, and who drew it.	Section 3.1
2	State whether the annotations were made independently, jointly, or by propagation and registration.	Section 3.1
3	Report performance against every available reference, not only one.	Section 4.3
4	Report the value against the least favourable reference alongside the others.	Section 4.3
5	Report the per-patient reference spread as a distribution.	Section 4.3
6	Where a multi-reader subset exists, report inter-reader agreement on the same cases as the scale against which the other numbers are read.	Section 4.1 and Section 4.2
7	Report boundary-sensitive and volumetric measures alongside overlap.	Section 4.3
8	State the registration and resampling method, and report how much agreement is lost to transferring masks between grids and how much changes with the transform.	Section 3.4 and Section 4.2

## Data Availability

The MRI dataset analysed in this study is publicly available on Zenodo (record 13131827, https://zenodo.org/records/13131827, accessed on 31 October 2026) under a CC BY 4.0 licence [43]. The analysis code, the fixed patient-level split and the nnU-Net fold definitions are publicly available at https://github.com/ema-pandilova/npc-multireference-eval (accessed on 31 October 2026).

## Supplementary Materials

The following supporting information can be downloaded at https://www.mdpi.com/article/10.3390/diagnostics16213632/s1. Figure S1: Image and mask grid mismatch in the two-dimensional export; Figure S2: Cross-sequence variability of the released morphological descriptors; Figure S3: Training and validation curves, and two training runs of the T1WI 3D model; Table S1: Scanner models recovered from the DICOM headers; Table S2: Cross-sequence spread of each released morphological descriptor and rank correlation with T stage; Table S3: Registration and grid transfer per sequence pair; Table S4: Model-level decomposition of the reference effect; and Table S5: The two-reader subset against the remaining patients.

## Abbreviations

The following abbreviations are used in this manuscript:

ASSD	Average symmetric surface distance
CE-T1WI	Contrast-enhanced T1-weighted imaging
CI	Confidence interval
DSC	Dice similarity coefficient
GPU	Graphics processing unit
HD95	95th percentile Hausdorff distance
ICC	Intraclass correlation coefficient
IQR	Interquartile range
MRI	Magnetic resonance imaging
RVE	Relative volume error
SD	Standard deviation
T1WI	T1-weighted imaging
T2WI	T2-weighted imaging
