# Supplementary material for: Multi-Reference Evaluation of Deep Learning Tumour Segmentation in Multi-Sequence Nasopharyngeal Carcinoma MRI
Source: Diagnostics (Basel). 2026 Nov 3;16(21):3632. doi: 10.3390/diagnostics16213632 (PMC13761546; doi:10.3390/diagnostics16213632)
Supplement: Supplementary file 1 [file diagnostics-16-03632-s001.zip › diagnostics-4635463-supplementary.pdf]

# Supplementary Materials: Multi-Reference Evaluation of Deep Learning Tumour Segmentation in Multi-Sequence Nasopharyngeal Carcinoma MRI

**Ema Pandilova [1,*], Marko Petrov [1], Ivan Chorbev [1], Ivan Kitanovski [1], Aleksandar Stojmenski [1] and Dimitar Trajanov [1]**

Sections S1 to S6 are cited from the main text. Sections S1 to S4 report secondary analyses, and Sections S5 and S6 give the per-pair and per-model detail behind the registration, resampling and reader analyses of the main text.

## S1. Audit of the Distributed Files

All 277 patients have all three sequences, comprising 22 745 image slices and 22 724 masks in the two-dimensional export; the 21 absent masks belong to two patients. The audit found five discrepancies between the distributed files and their documentation. None affects the three-dimensional data on which every analysis of the main text rests, and we report them so that users of the files can correct for them.

The first and most consequential concerns the two-dimensional TIFF export. Its images are uniformly 256 $\times$ 256, whereas its masks keep the native acquisition matrix of 432, 448, 480, 512, 528, 560, 576 or 640 pixels square. Of 22 724 image and mask pairs, 21 143 (93.0%) are mismatched, and all 277 patients are affected; T1WI and CE-T1WI pairs are mismatched in 100% of cases and T2WI pairs in 80.1%, the exception being patients whose native T2WI matrix was already 256. Pairing the files by name, which is the natural thing to do, associates each image with a mask on a different grid, and an overlay places the contour outside the head (Figure S1). The conversion utility distributed with the dataset explains the pattern: it resizes images to a default of 256 pixels and writes mask slices from the NIfTI volumes without resizing. The mismatch is fully recoverable by nearest-neighbour resampling of each mask onto its image grid, the only interpolation that preserves a binary label. The two-dimensional archive is not described in the data descriptor, and its files carry no spatial calibration and are 8-bit, so volumetry in physical units requires the three-dimensional data.

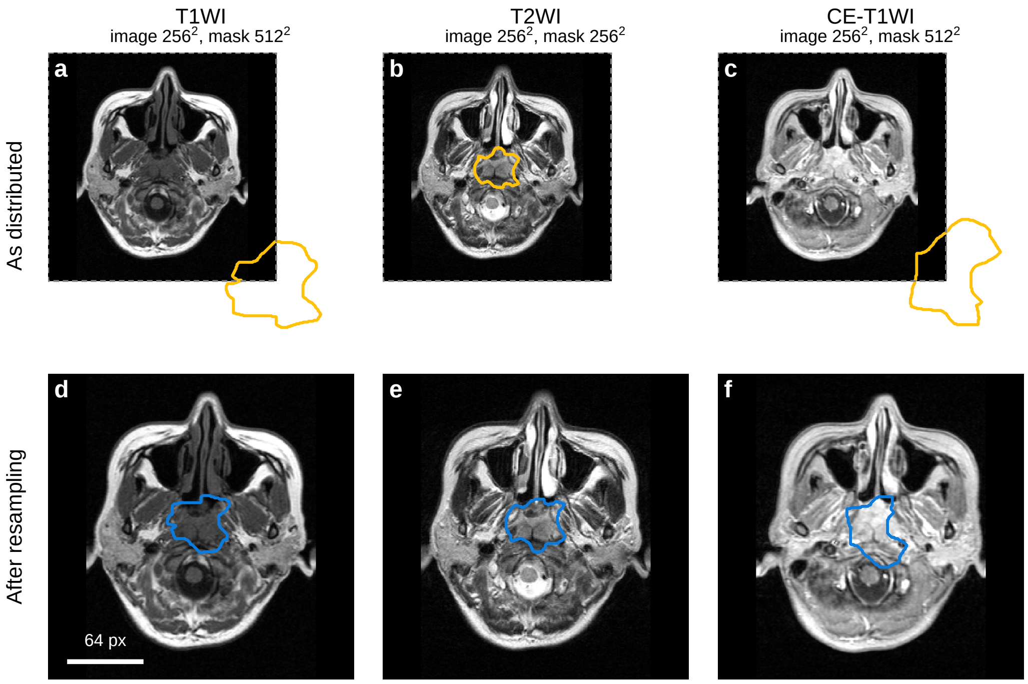


**Figure S1.** Image and mask grid mismatch in the two-dimensional export, one patient. (**a**–**c**) Masks in their own coordinate system, as pairing by filename produces; dashed square, the image's own grid. (**d**–**f**) After nearest-neighbour resampling of the mask onto the image grid.

Second, the data descriptor states that the two-reader archive contains 256 × 256 images and masks, whereas only 16 of its 90 masks have that size. Third, the descriptor names a metadata file as the source of scanner information, but the file is absent from the released archive; acquisition parameters were therefore recovered from the DICOM headers of all 831 series, and the recovered per-model patient counts reproduce the descriptor's own table. Fourth, both released tables are semicolon-delimited and carry a decorative first row, and the morphology table has a two-level header, so that a default reader returns a single malformed column. Fifth, the descriptor's body text cites Zenodo record 10900202, which is restricted; the open CC BY 4.0 record is 13131827.

The clinical table has minor coding inconsistencies (one stage value differing only in letter case, one Epstein–Barr virus DNA value written with full-width parentheses, and one tumour coded T0 in a primary carcinoma cohort). Progression-free survival is missing for 26 patients and is recorded as a time without an event or censoring indicator, which is why no time-to-event analysis is reported.

## S2. Acquisition Hardware and Cross-Sequence Variability

Acquisition metadata recovered from the DICOM headers show six scanner models from three vendors (Table S1). Field strength, which the dataset publication does not report, spans a factor of three: 93 patients (33.6%) were imaged at approximately 0.95 T, 171 (61.7%) at 1.5 T and 13 (4.7%) at 3.0 T.

**Table S1.** Scanner models recovered from the DICOM headers.

| Vendor | Model | Field strength (T) | Patients |
|---|---|---|---|
| Philips | Achieva | 1.5 | 146 |
| Siemens | MAGNETOM IMPACT | 0.95 | 93 |
| GE | Signa HDxt | 1.5 | 20 |
| GE | DISCOVERY MR750w | 3.0 | 12 |
| GE | OPTIMA MR360 | 1.5 | 5 |
| GE | SIGNA EXCITE | 3.0 | 1 |

The cross-sequence volume spread varied with field strength, with medians of 17.4% at 0.95 T, 20.6% at 1.5 T and 27.4% at 3.0 T (Kruskal–Wallis $H = 13.7$, $p = 1.1 \times 10^{-3}$). A test on vendor is nominally significant as well ($H = 10.5$, $p = 5.4 \times 10^{-3}$) but is not an independent finding, because the 0.95 T examinations are exactly the Siemens ones and every 3.0 T examination is on a GE system. The direction is the opposite of the expectation that a weaker magnet would be noisier; one reading is that higher field strength and finer effective resolution reveal more sequence-specific detail at the tumour margin. The analysis is exploratory. Only 13 patients were imaged at 3.0 T, and within one institution field strength is confounded with vendor, scanner model and acquisition period, so no causal attribution to any of them is possible from these data.

## S3. Prediction of Sequence-Unstable Patients

Because about half of the patients show a cross-sequence volume spread above 20%, we tested whether they can be identified in advance from a single sequence. The target was a spread above 20% (prevalence 49.5%). Predictors were the morphology of one sequence together with acquisition metadata and stage, the information available when only that sequence has been read. Logistic regression and gradient boosting were evaluated with repeated stratified five-fold cross-validation.

Prediction was poor. Logistic regression reached an area under the receiver operating characteristic curve of 0.62, 0.63 and 0.61 with T1WI, T2WI and CE-T1WI available respectively, and gradient boosting 0.55, 0.53 and 0.59. At an operating point that catches about 80% of unstable cases, 71% to 74% of all patients would have to be flagged. Field strength was the strongest single predictor, consistent with Section S2. We report this as a negative result: sequence dependence could not be screened for from the information available, which leaves reporting the sequence as the practical remedy.

## S4. Secondary Morphology Analyses

The four released shape descriptors were not equally affected by the choice of sequence (Table S2, Figure S2). Volume was the least reproducible across sequences and maximum diameter the most, with median spreads differing by a factor of 2.6. A lower spread does not by itself imply more stable classification: when patients were assigned to tertiles, maximum diameter reassigned slightly more patients than volume (64 against 55 of 277), because its narrower range across patients places the tertile boundaries closer together. Reproducibility and discriminative range trade off against each other, and a descriptor cannot be recommended on reproducibility alone.

**Table S2.** Cross-sequence spread (range divided by mean) of each released morphological descriptor, and rank correlation with T stage ($n = 277$).

| Descriptor | Cross-sequence spread | | | | Against T stage | |
|---|---|---|---|---|---|---|
| | Median (%) | IQR (%) | Max (%) | Friedman $p$ | $\rho$ | $p$ |
| Volume | 20.0 | 12.5–30.2 | 146.4 | $4.9 \times 10^{-38}$ | 0.440 | $1.5 \times 10^{-14}$ |
| Surface area | 16.0 | 10.7–23.0 | 135.2 | $3.4 \times 10^{-46}$ | 0.399 | $5.6 \times 10^{-12}$ |
| Surface regularity | 9.7 | 5.9–14.3 | 73.3 | $9.9 \times 10^{-8}$ | 0.061 | 0.31 |
| Maximum diameter | 7.7 | 4.6–12.5 | 42.1 | $1.7 \times 10^{-20}$ | 0.383 | $4.0 \times 10^{-11}$ |

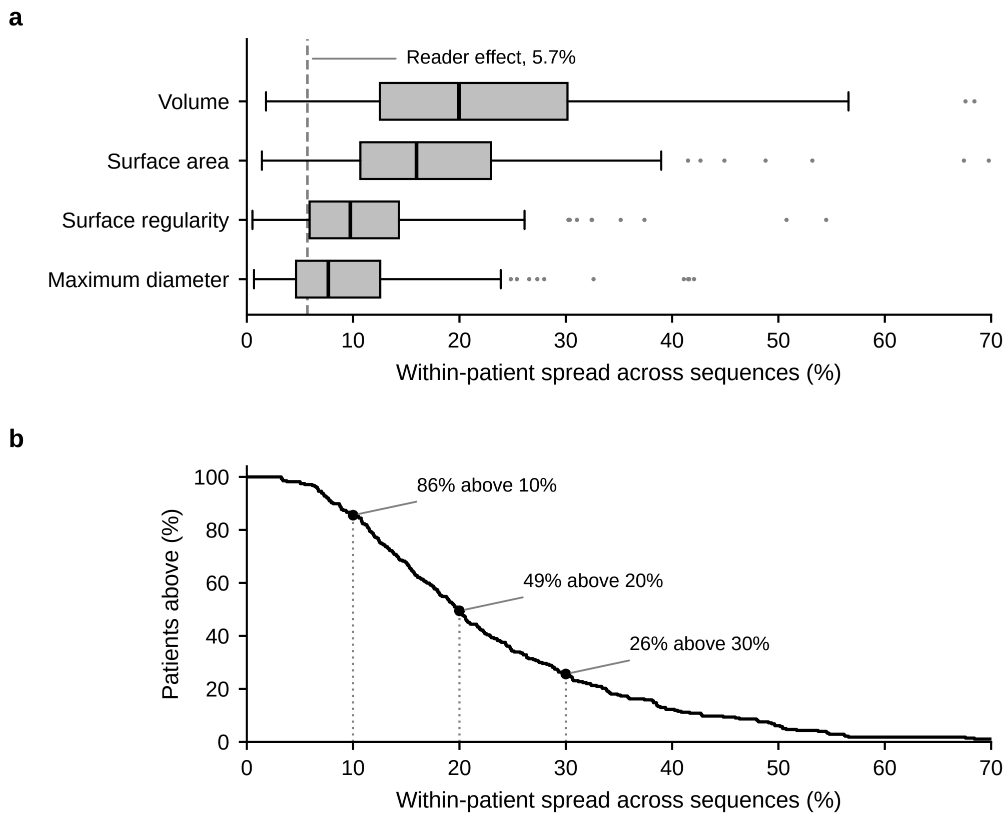


**Figure S2.** Cross-sequence variability of the released morphological descriptors ($n = 277$). (**a**) Within-patient spread per descriptor; the dashed line marks the 5.7% pairwise reader effect for orientation only, since the spreads are ranges over three sequences and are larger than a pairwise difference by construction. Five of 1108 values exceed the axis, the largest 146%. (**b**) Patients exceeding a given volume spread.

## S5. Registration and Resampling per Sequence Pair

The main text summarises the registration and grid-transfer analysis across all sequence pairs. Table S3 gives it per pair, together with agreement when the moving reference is placed by scanner coordinates alone, without registration, and two distances that describe the registration itself: how far the rigid registration moved the tumour centroid away from its scanner-coordinate position, and how far apart the rigid and affine registrations placed it.

**Table S3.** Registration and grid transfer per sequence pair (medians, IQR in brackets). Header, placement by scanner coordinates; affine values for converged pairs; correction, rigid shift of the tumour centroid from its scanner position.

| Pair | *n* | Dice, rigid | Header | Affine | Affine diverged | Floor | NN | Correction (mm) | Rigid–affine (mm) |
|---|---|---|---|---|---|---|---|---|---|
| T1WI vs T2WI | 275 | 0.806 (0.753–0.841) | 0.807 | 0.803 | 4 | 0.979 (0.961–0.990) | 0.995 | 0.64 | 0.36 |
| CE-T1WI vs T1WI | 276 | 0.773 (0.714–0.828) | 0.747 | 0.778 | 42 | 0.931 (0.861–0.973) | 1.000 | 1.81 | 0.42 |
| CE-T1WI vs T2WI | 276 | 0.759 (0.694–0.815) | 0.743 | 0.781 | 43 | 0.933 (0.860–0.968) | 0.998 | 2.11 | 0.77 |
| All pairs | 827 | 0.783 (0.722–0.830) | 0.772 | 0.791 | 89 | 0.955 (0.889–0.983) | 0.998 | 1.34 | 0.47 |

Three observations qualify the summary in the main text. First, the rigid registration failed in five pairs: CE-T1WI against T2WI in patient 055, CE-T1WI against T1WI and against T2WI in patient 076, and CE-T1WI against T2WI and T1WI against T2WI in patient 176. Each moved the tumour 22.6 to 130.8 mm from its scanner-coordinate position, whereas scanner coordinates alone gave agreement of 0.628 to 0.865. The registration was initialised by aligning the geometric centres of the two volumes, and where two sequences cover different fields of view that starting point lies far from the true alignment. Registration was nonetheless essential in other patients: in patient 151 the rigid registration moved the tumour by 16.9 and 14.9 mm and raised agreement from 0.094 to 0.829 and from 0.079 to 0.643, relative to scanner coordinates, so neither scanner coordinates nor the registration can be trusted without a check.

Second, the affine registration diverged in 89 pairs, 85 of them with CE-T1WI as the fixed image, and the affine column of Table S3 summarises the pairs where it converged. Third, the grid-transfer floor depends on whether the two sequences share slice planes. In 731 pairs they did, and the floor was 0.961 (IQR 0.905 to 0.984); in the 96 pairs acquired on offset or differently spaced planes it was 0.888 (IQR 0.809 to 0.933), and the observed cross-sequence agreement was correspondingly lower (0.721 against 0.786). Offset planes occurred in 16.7% of the pairs involving CE-T1WI and in 1.5% of the pairs of unenhanced sequences.

## S6. Model-Level Components per Model

Table S4 decomposes, for each trained 3D model and each other sequence, the drop from the model's score against its own reference to its score against that sequence's reference. The own reference is first passed through the other sequence's grid; the change in score is the grid-transfer loss, and what remains is the part attributable to the reference together with residual registration error.

**Table S4.** Model-level decomposition of the reference effect (102 patients). Loss is the change in score when the model's own reference passes through the other grid; the last column compares two scores that share the same grid transfer.

| Model | Other grid | Own, native | Own through grid | Grid-transfer loss: Median | Grid-transfer loss: Mean | Grid-transfer loss: % over 0.02 | Other reference | Observed drop | Beyond grid transfer (95% CI) |
|---|---|---|---|---|---|---|---|---|---|
| T1WI | T2WI | 0.726 | 0.728 | −0.000 | 0.002 | 11 | 0.685 | 0.035 | 0.038 (0.020 to 0.051) |
| T1WI | CE-T1WI | 0.726 | 0.699 | 0.001 | 0.024 | 25 | 0.673 | 0.045 | 0.036 (0.022 to 0.047) |
| T2WI | T1WI | 0.723 | 0.715 | 0.000 | 0.008 | 15 | 0.702 | 0.030 | 0.026 (0.018 to 0.032) |
| T2WI | CE-T1WI | 0.723 | 0.709 | 0.000 | 0.032 | 27 | 0.679 | 0.042 | 0.035 (0.021 to 0.054) |
| CE-T1WI | T1WI | 0.744 | 0.706 | 0.003 | 0.025 | 33 | 0.693 | 0.043 | 0.030 (0.020 to 0.044) |
| CE-T1WI | T2WI | 0.744 | 0.714 | 0.004 | 0.025 | 30 | 0.669 | 0.056 | 0.048 (0.033 to 0.060) |

The grid-transfer loss is near zero in most patients and concentrated in a minority, more often where CE-T1WI is involved, so its median alone understates it; the table therefore also gives its mean and the share of patients in whom it exceeds 0.02. The beyond-grid-transfer column is the comparison that carries the same grid transfer on both sides, and it is

positive for every model and sequence, with every confidence interval excluding zero. The three registrations that failed quality control (patient 169, T1WI and T2WI models against CE-T1WI; patient 234, T2WI model against CE-T1WI) are included here, as in the primary analysis of the main text, which also reports the results without them. Neither patient was annotated by both radiologists, so the reader-controlled comparison of Table 6 in the main text involves none of these registrations.

## S7. Representativeness of the Two-Reader Subset

Thirty of the 277 patients were annotated independently by both radiologists. The subset was fixed by the data providers and the data descriptor states no selection criterion, so how it was chosen cannot be recovered from the released files. Its identifiers are not consistent with a simple random draw: twenty of the thirty are numbered 045 or below, and the remaining ten run from 103 to 274. A random draw of thirty from 277 would be expected to place about five in the lower range, and the observed twenty gives a hypergeometric $p < 10^{-9}$.

Table S5 compares the subset with the other 247 patients on the two characteristics the released clinical table supports. No difference in tumour volume is detected on any of the three sequences. Thirty patients against 247 gives little power to detect one, so this is not evidence that the two groups match, and the subset's upper quartile is the lower of the two on every sequence, by a factor of 1.4 to 1.5, so its largest tumours are the less well represented. T category is less balanced: the subset holds a higher proportion of T1 and T2 disease, 47% against 26%, although the full four-category distribution does not differ and the nominal Fisher value of 0.031 would rise to 0.15 under Holm correction for the five comparisons in this table, so the imbalance is a caution rather than an established difference. Taken together the subset is not established to be representative, and the reader effect reported in the main text is best read as measured in patients whose disease is probably somewhat earlier, and whose largest tumours are less well represented, than the cohort as a whole.

**Table S5.** The thirty double-annotated patients against the remaining 247. Volume is given as median [IQR] and compared with the Mann–Whitney $U$ test; T category is compared with Fisher's exact test on the early against advanced collapse. One patient outside the subset is coded T0 and is excluded from the T comparisons.

| Characteristic | Two-reader subset ($n = 30$) | Other patients ($n = 247$) | $p$ |
|---|---|---|---|
| T1 or T2 | 14 (46.7%) | 65 (26.4%) | 0.031 |
| T3 or T4 | 16 (53.3%) | 181 (73.6%) | |
| T1 | 2 | 13 | 0.12 |
| T2 | 12 | 52 | |
| T3 | 10 | 106 | |
| T4 | 6 | 75 | |
| Volume, T1WI ($cm^3$) | 7.72 [4.93, 12.45] | 8.69 [4.83, 18.30] | 0.27 |
| Volume, T2WI ($cm^3$) | 7.07 [4.76, 12.20] | 8.99 [4.62, 17.11] | 0.32 |
| Volume, CE-T1WI ($cm^3$) | 8.11 [5.55, 13.85] | 9.74 [5.57, 19.24] | 0.23 |

## S8. Training and Validation Curves

Figure S3 gives the training and validation loss of every configuration reported in the main text, and the validation pseudo-Dice of the two T1WI 3D runs that differ only in random initialisation and data order. The validation loss of each configuration settles by roughly epoch 100 and stays flat thereafter while the training loss continues to fall slowly, which is the expected separation for this amount of data and shows no collapse of the validation signal. The two T1WI runs track each other throughout, differing by a mean of 0.020 in pseudo-Dice over the last fifty epochs and by 0.013 at the final epoch.

The quantity in panel (b) is nnU-Net's internal validation pseudo-Dice, accumulated over patches during training on its own validation split. It is not the held-out score reported in the main text, and the two are not comparable in value.

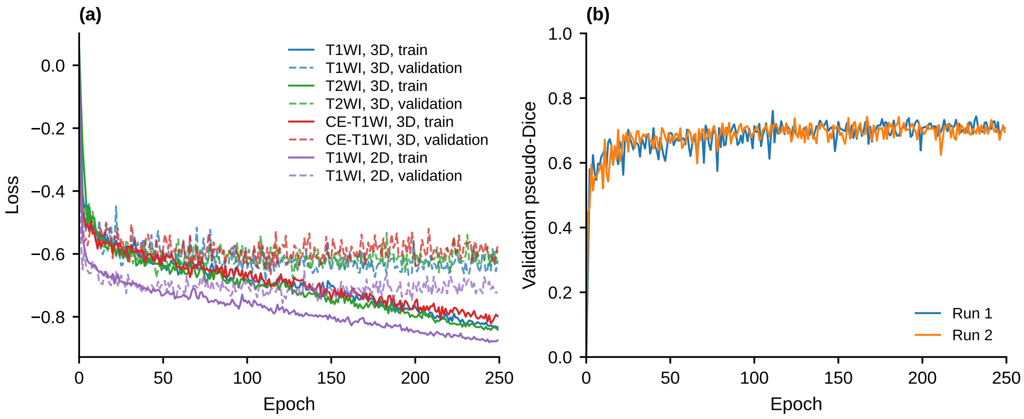


**Figure S3.** (**a**) Training loss (solid) and validation loss (dashed) against epoch for the four trained configurations. (**b**) Validation pseudo-Dice against epoch for the two training runs of the T1WI 3D model.
